# 

**Published:** 1849-04

**Authors:** 


					APRIL, 1-49
THE
OF
w m "
cxt\
:*p u
;? . -r. '
L -
PUBLISHED trin$Eft THE AUSPICES OF TAB
Celof Bental ,
E diioes:
CHAPIN A. HARRIS, M. D., D. D. S.
WESTCOTT, M. D., D. D. S.
D WINELLE, D. D. S,
CORRESPONDENT ftf* ENGLAND :
D. ?>.,7 Gower-st. Bedford Square,
BALTIMORE:
BBRRY,
PHILADELPHIA: LINDSAY & BLAKISTON.
mm
. ^..--g??? ? : m ?? ?
9500 i?er payable In ndvaoce.
1
This Pel

				

